# Supplementary material for: Locking the lattice oxygen in RuO2 to stabilize highly active Ru sites in acidic water oxidation
Source: Nat Commun. 2024 Mar 20;15:2501. doi: 10.1038/s41467-024-46815-6 (PMC10954744; doi:10.1038/s41467-024-46815-6)
Supplement: Supplementary file 1 — Supplementary Information [file 41467_2024_46815_MOESM1_ESM.pdf]

Locking the lattice oxygen in RuO_2 to stabilize highly active Ru sites in acidic water oxidation

Xinyu Ping[1,3], Yongduo Liu[1,3], Lixia Zheng[1], Yang Song[1], Lin Guo[2], Siguo Chen[1]*, Zidong Wei[1]

[1] College of Chemistry and Chemical Engineering, Chongqing University, Chongqing, China

[2] State Key Laboratory of Catalytic Materials and Reaction Engineering, SINOPEC Research Institute of Petroleum Processing Co., Ltd., Beijing, China

[3] These authors contributed equally to this work.

* Correspondence to: csg810519@126.com

Supplementary Information for

Locking the lattice oxygen in RuO_2 to stabilize highly active Ru sites in acidic water oxidation

Contents:

Supplementary Figure 1. (a) TEM image of Si-RuO_2-0; (b) particle size distribution of Si-RuO_2-0; (c) HRTEM image of Si-RuO_2-0; (d) HAADF-STEM and corresponding elemental mapping images of Ru and O........4

Supplementary Figure 2. (a) TEM image of Si-RuO_2-0.05; (b) particle size distribution of Si-RuO_2-0.05; (c) HRTEM image of Si-RuO_2-0.05; (d) HAADF-STEM and corresponding elemental mapping images of Si, Ru and O. 5

Supplementary Figure 3. (a) TEM image of Si-RuO_2-0.1; (b) particle size distribution of Si-RuO_2-0.1; (c) HRTEM image of Si-RuO_2-0.1; (d) HAADF-STEM and corresponding elemental mapping images of Si, Ru and O. 6

Supplementary Figure 4. (a) TEM image of Si-RuO_2-0.2; (b) particle size distribution of Si-RuO_2-0.2; (c) HRTEM image of Si-RuO_2-0.2; (d) HAADF-STEM and corresponding elemental mapping images of Si, Ru and O. 7

Supplementary Figure 5. (a) TEM image of Si-RuO_2-0.3; (b) particle size distribution of Si-RuO_2-0.3; (c) HRTEM image of Si-RuO_2-0.3; (d) HAADF-STEM and corresponding elemental mapping images of Si, Ru and O. 8

Supplementary Figure 6. The first derivative curves of the Ru *K*-edge XANES spectra of Si-RuO_2-0.1 and Com-RuO_2........9

Supplementary Figure 7. EXAFS fitting in R-space of (a) Si-RuO_2-0.1 and (b) Com-RuO_2. R-mag represents the magnitude of the EXAFS data in R-space; R-Imag is the imaginary part of the EXAFS data in R-space........10

Supplementary Figure 8. The modeled structures of (a) $Ru_{16}O_{32}$ and (b-h) different $Ru_{16}Si_2O_{32}$ after optimization (denoted A-M-X, where A represents the first Si atom occupies the A-site; M represents the position occupied by the second Si atom, M = A, B, C or D; X represents the spatial position located by second Si atom, X = t or b, t is top, b is bottom). Color code: Si (blue), Ru (gray) and O (red)........11

Supplementary Figure 9. Gibbs free energy of different structural models. The A-C-b model as most stable structure was selected for subsequent analysis. 12

Supplementary Figure 10. PDOS plots of the Ru 4*d* and O 2*p* orbitals of (a) Si-RuO_2 slab and RuO_2 slab and (b) Si-RuO_2 bulk and RuO_2 bulk........13

Supplementary Figure 11. Theoretical calculations of the acidic OER activity of the established models of on (a) Si-RuO_2 and (b) RuO_2........14

Supplementary Figure 12. S-number of Si-RuO_2-0.1, Si-RuO_2-0 and Com-RuO_2 at 10 mA cm^{-2}. 15

Supplementary Figure 13. TEM image of Si-RuO_2-0.1 after the 800 h test....16

Supplementary Figure 14. Ru 3*p* spectra for (a) Si-RuO_2-0.1, (b) Si-RuO_2-0 and (c) Com-RuO_2 before and after stability testing....17

Supplementary Figure 15. O 1*s* spectra for (a) Si-RuO_2-0.1, (b) Si-RuO_2-0 and (c) Com-RuO_2 before and after stability testing....18

Supplementary Figure 16. DEMS measurements of (a) Si-RuO_2-0.1 and (b) Com-RuO_2....19

Supplementary Table 1. Structural parameters of Si-RuO_2-0.1 and the reference samples extracted from the EXAFS fitting....20

Supplementary Table 2. Lattice parameters and unit-cell volume of the modeled structures after optimization. 21

Supplementary Table 3. The detailed information for the free energy change of reaction intermediates (*OH, *O, *OOH) on the unsaturated Ru site. 22

Supplementary Table 4. Comparison of the OER performance of reported representative Ru-based oxide electrocatalysts in acidic media. 23

Supplementary Note 1 24

Supplementary References 25

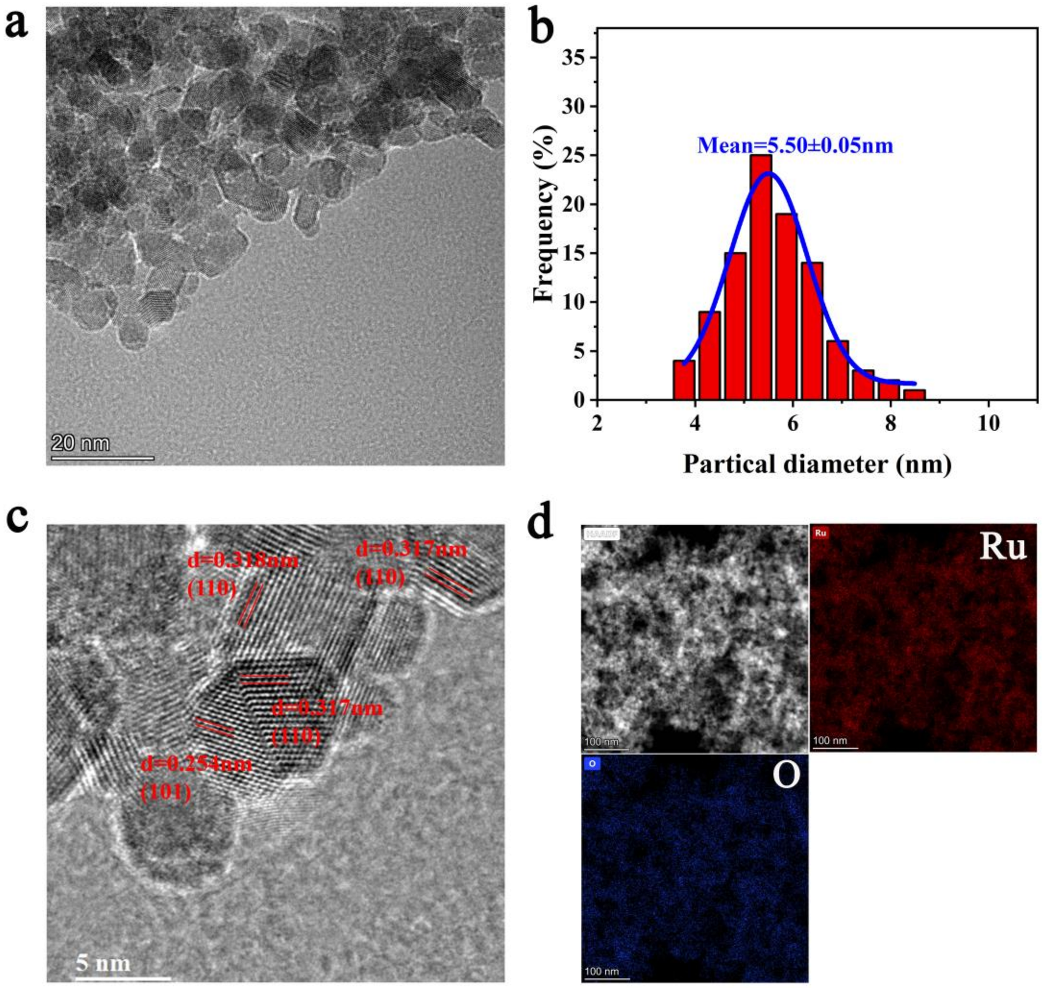

Supplementary Figure 1. (a) TEM image of Si-RuO_2-0; (b) particle size distribution of Si-RuO_2-0; (c) HRTEM image of Si-RuO_2-0; (d) HAADF-STEM and corresponding elemental mapping images of Ru and O.

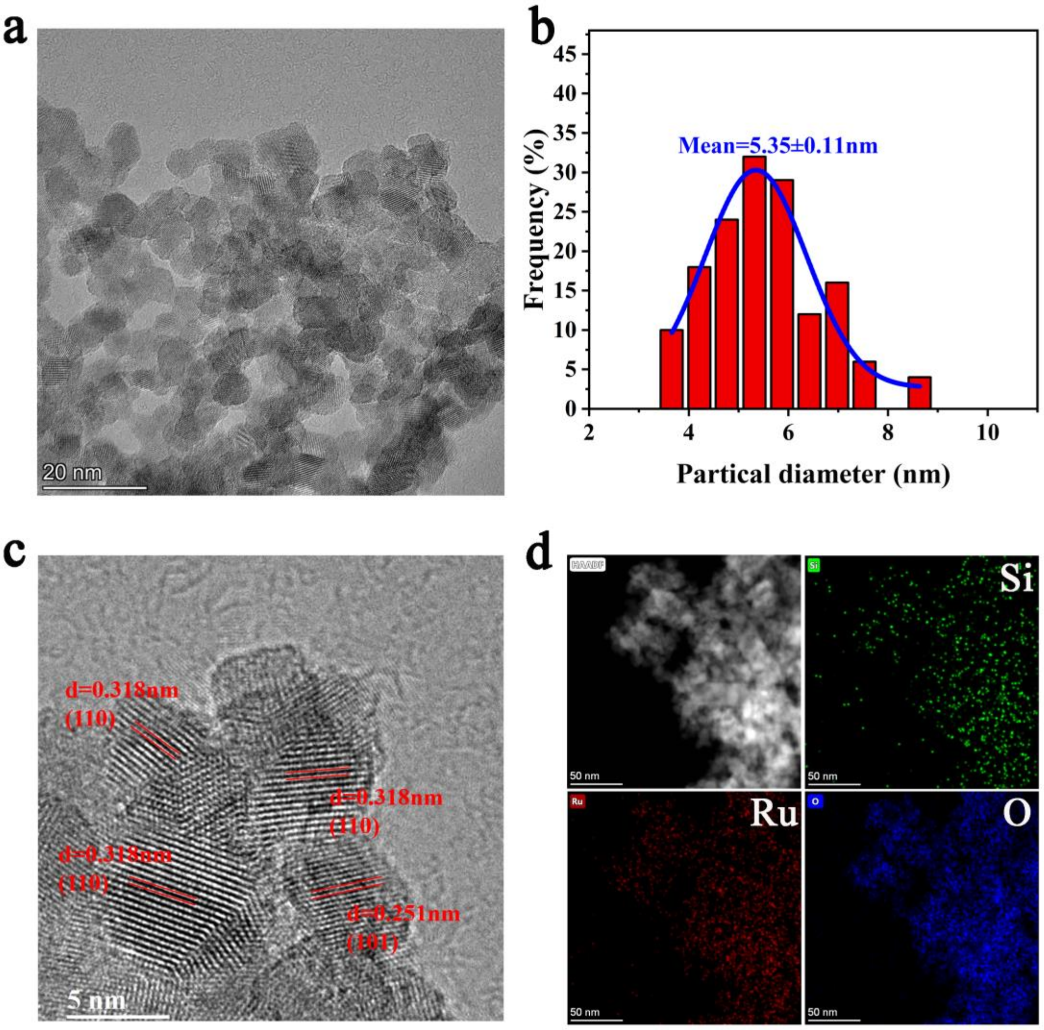

Supplementary Figure 2. (a) TEM image of Si-RuO_2-0.05; (b) particle size distribution of Si-RuO_2-0.05; (c) HRTEM image of Si-RuO_2-0.05; (d) HAADF-STEM and corresponding elemental mapping images of Si, Ru and O.

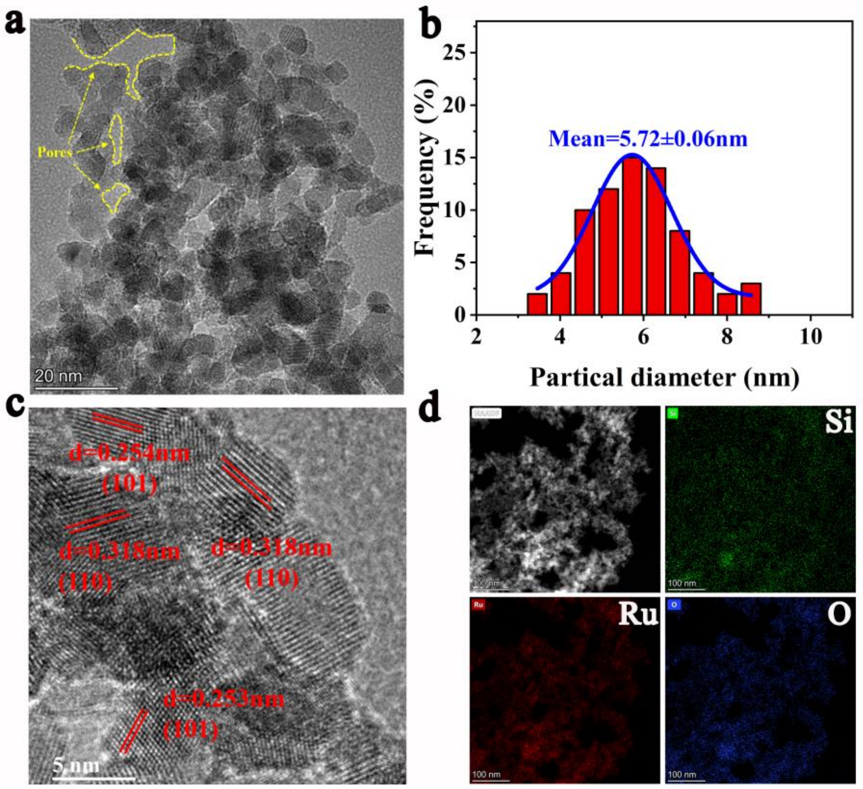

Supplementary Figure 3. (a) TEM image of Si-RuO_2-0.1; (b) particle size distribution of Si-RuO_2-0.1; (c) HRTEM image of Si-RuO_2-0.1; (d) HAADF-STEM and corresponding elemental mapping images of Si, Ru and O.

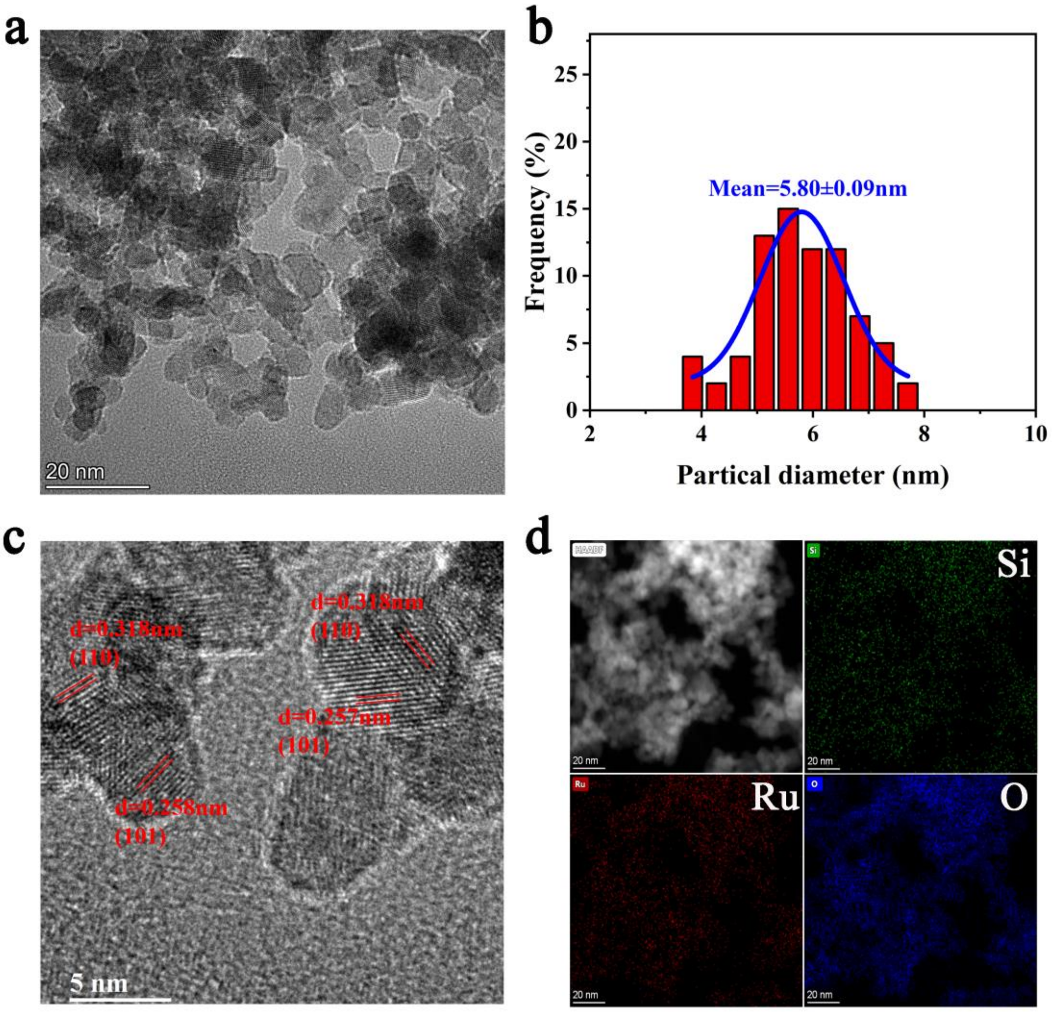

Supplementary Figure 4. (a) TEM image of Si-RuO_2-0.2; (b) particle size distribution of Si-RuO_2-0.2; (c) HRTEM image of Si-RuO_2-0.2; (d) HAADF-STEM and corresponding elemental mapping images of Si, Ru and O.

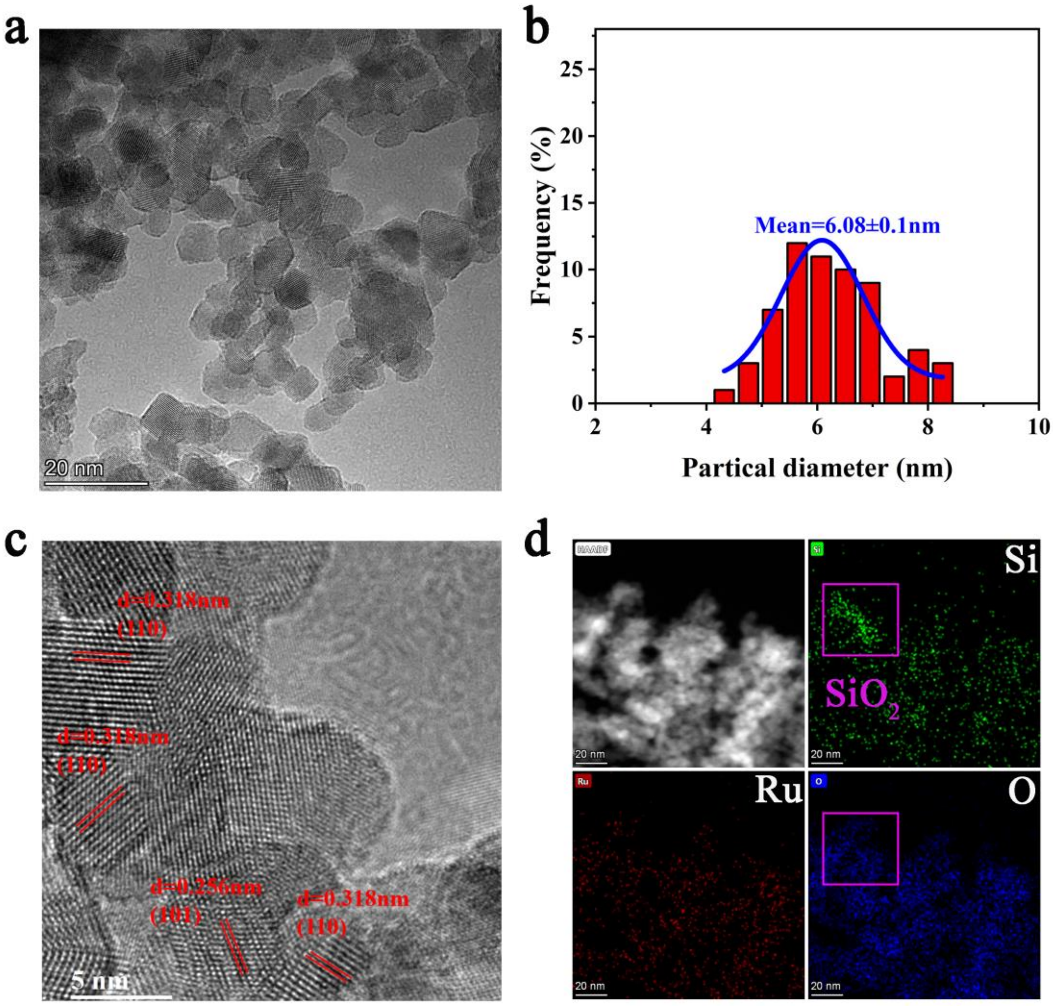

Supplementary Figure 5. (a) TEM image of Si-RuO_2-0.3; (b) particle size distribution of Si-RuO_2-0.3; (c) HRTEM image of Si-RuO_2-0.3; (d) HAADF-STEM and corresponding elemental mapping images of Si, Ru and O.

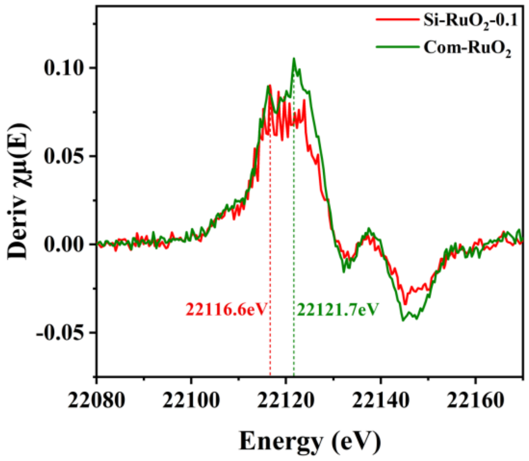

Supplementary Figure 6. The first derivative curves of the Ru *K*-edge XANES spectra of Si-RuO_2-0.1 and Com-RuO_2.

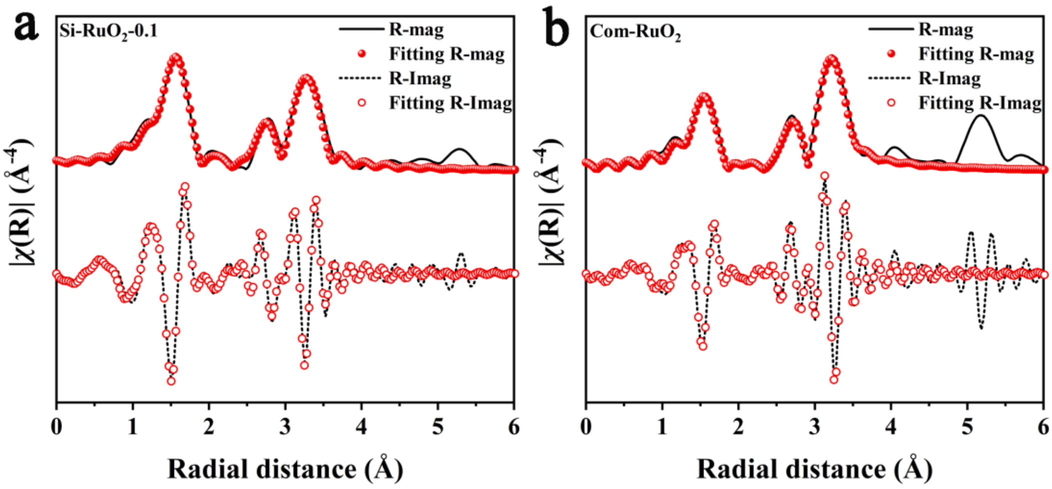

Supplementary Figure 7. EXAFS fitting in R-space of (a) Si-RuO_2-0.1 and (b) Com-RuO_2. R-mag represents the magnitude of the EXAFS data in R-space; R-Imag is the imaginary part of the EXAFS data in R-space.

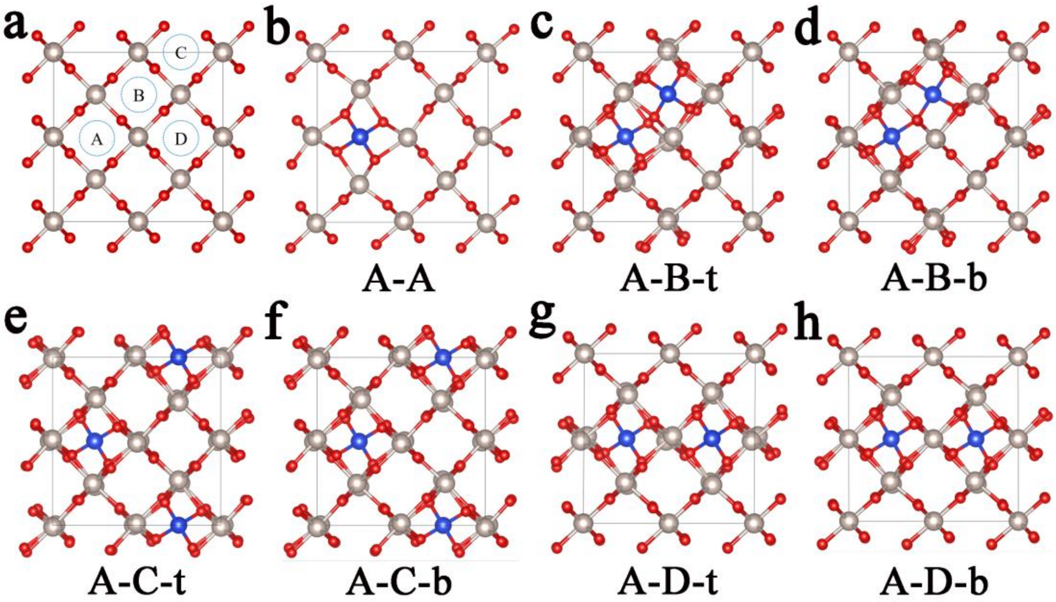

Supplementary Figure 8. The modeled structures of (a) $Ru_{16}O_{32}$ and (b-h) different $Ru_{16}Si_2O_{32}$ after optimization (denoted A-M-X, where A represents the first Si atom occupies the A-site; M represents the position occupied by the second Si atom, M = A, B, C or D; X represents the spatial position located by second Si atom, X = t or b, t is top, b is bottom). Color code: Si (blue), Ru (gray) and O (red).

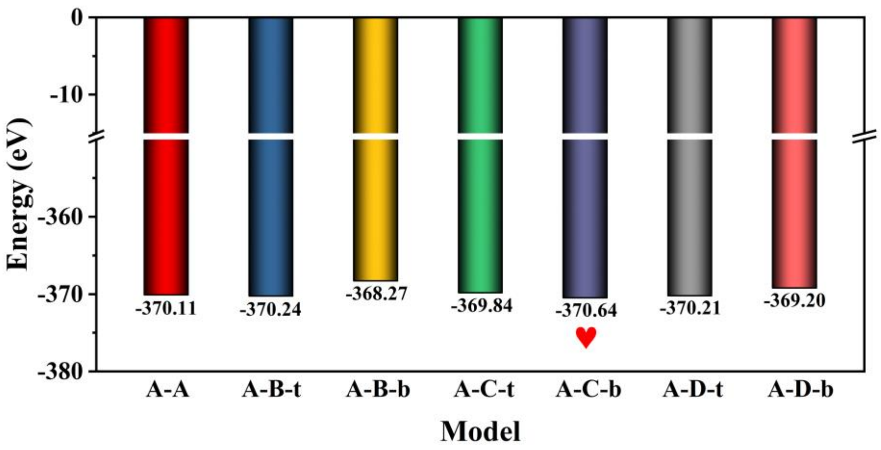

Supplementary Figure 9. Gibbs free energy of different structural models. The A-C-b model as most stable structure was selected for subsequent analysis.

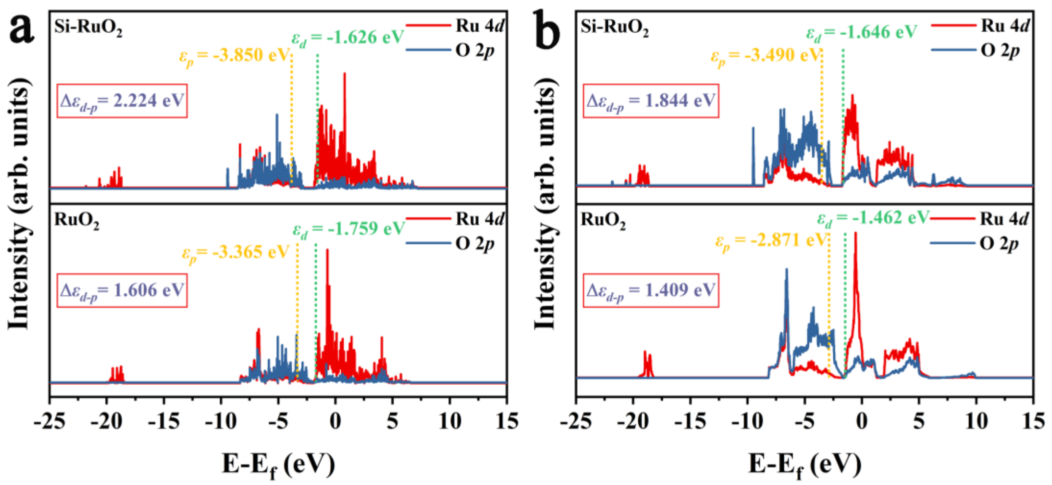

Supplementary Figure 10. PDOS plots of the Ru 4*d* and O 2*p* orbitals of (a) Si-RuO_2 slab and RuO_2 slab and (b) Si-RuO_2 bulk and RuO_2 bulk.

Although the band center of Ru 4*d* on the bulk and the (110) plane present different trends, the gap between Ru 4*d* band center and O 2*p* band center is still enlarged due to the downshift of the O 2*p* band center. All results indicate that the covalency of Ru-O bond in Si-RuO_2 is lower than that of RuO_2.

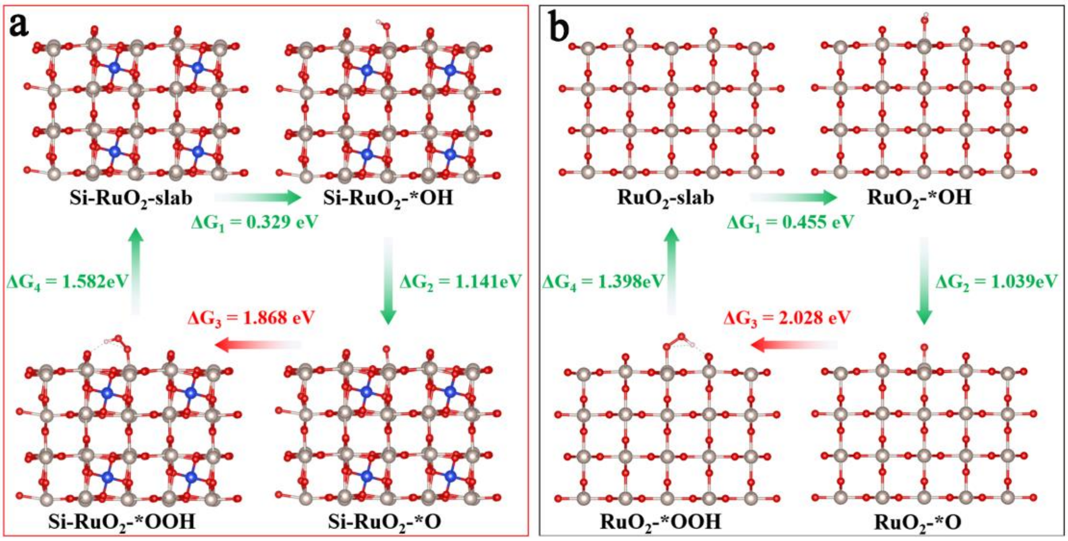

Supplementary Figure 11. Theoretical calculations of the acidic OER activity of the established models of on (a) Si-RuO_2 and (b) RuO_2.

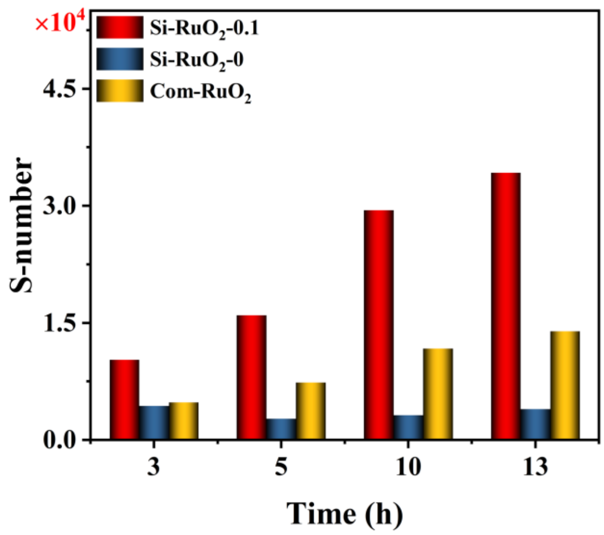

Supplementary Figure 12. S-number of Si-RuO_2-0.1, Si-RuO_2-0 and Com-RuO_2 at 10 mA cm^{-2}.

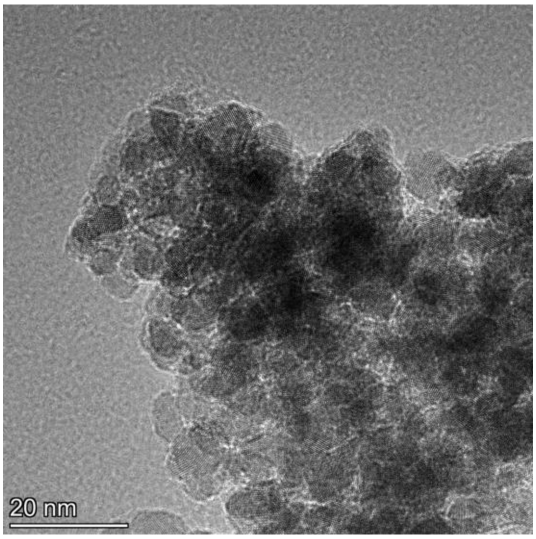

Supplementary Figure 13. TEM image of Si-RuO_2-0.1 after the 800 h test.

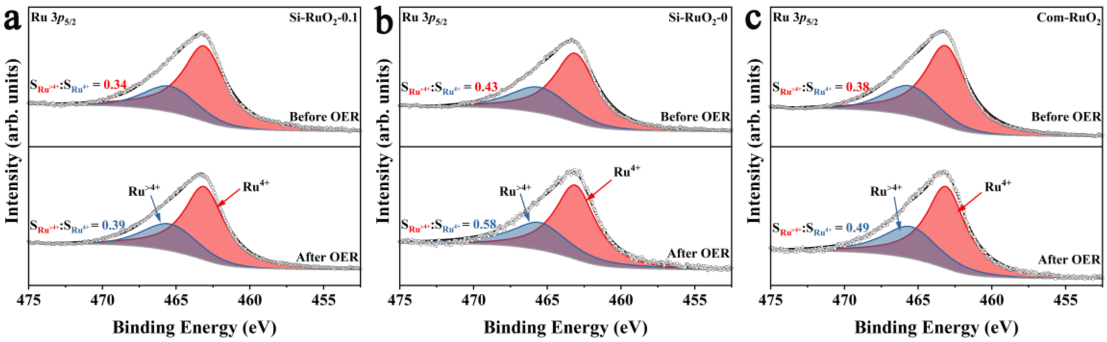

Supplementary Figure 14. Ru 3p spectra for (a) Si-RuO_2-0.1, (b) Si-RuO_2-0 and (c) Com-RuO_2 before and after stability testing.

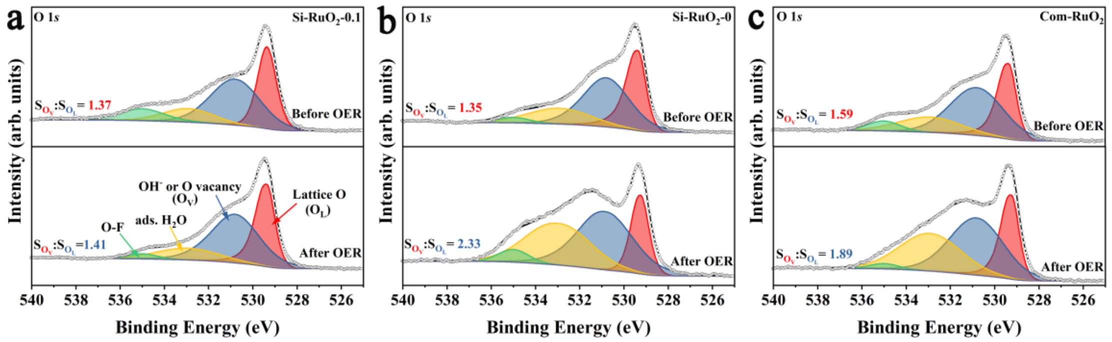

Supplementary Figure 15. O 1*s* spectra for (a) Si-RuO_2-0.1, (b) Si-RuO_2-0 and (c) Com-RuO_2 before and after stability testing.

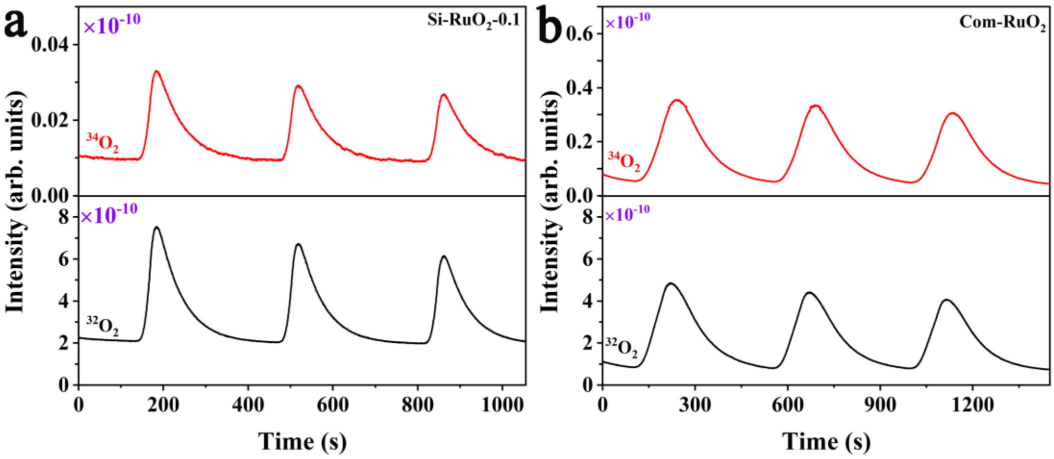

Supplementary Figure 16. DEMS measurements of (a) Si-RuO_2-0.1 and (b) Com-RuO_2.

Supplementary Table 1. Structural parameters of Si-RuO_2-0.1 and the reference samples extracted from the EXAFS fitting.

	Shell	CN	R(Å)	σ^2	ΔE_0 (eV)	R factor
Ru foil	Ru-Ru_1	6	2.62±0.008	0.00085	4.68±0.98	0.0029
	Ru-Ru_2	6	2.71±0.012	0.00005	-3.56±1.91	
Com-RuO_2	Ru-O	6	1.96±0.004	0.0027	-3.79±1.15	0.0143
	Ru-Ru_1	2	3.11±0.005	0.0025	-3.79±1.15	
	Ru-Ru_2	8	3.55±0.004	0.0030	-3.79±1.15	
Si-RuO_2-0.1	Ru-O	6.13±0.90	1.98±0.01	0.0047	4.68±3.45	0.0187
	Ru-Ru_1	1.26±0.32	3.13±0.03	0.0034	0.32±7.74	
	Ru-Ru_2	5.03±0.32	3.57±0.01	0.0045	3.10±2.81	

CN: coordination number; *R*: bond distance; σ^2: Debye-Waller factor; ΔE_0: inner potential correction. *R* factor: goodness of fit. S_0^2 was set to 0.92, according to the experimental EXAFS fit of the Ru foil reference by fixing *CN* as the known crystallographic value.

Supplementary Table 2. Lattice parameters and unit-cell volume of the modeled structures after optimization.

Modeled structures	Lattice parameters						Unit-cell volume（Å^3）
	**a**	**b**	**c**	**α**	**β**	**γ**	
$Ru_{16}O_{32}$	9.03983	9.03983	6.23868	90	90	90.0021	509.8158
A-A	9.31716	9.31966	6.25462	90.0054	89.9996	89.9945	543.1056
A-B-t	9.28840	9.28903	6.28375	89.9643	90.0362	90.0157	542.1636
A-B-b	9.22747	9.24128	6.32503	89.9737	90.1670	90.0108	539.3540
A-C-t	9.27393	9.27411	6.30608	90.0035	90.0035	90.0022	542.3695
A-C-b	9.28328	9.28311	6.29862	89.9967	90.0003	89.9999	542.8007
A-D-t	9.30198	9.25337	6.29996	89.9988	90.0006	89.5885	542.2534
A-D-b	9.38953	9.12285	6.31181	89.9984	90.0018	90.0746	540.6645

Supplementary Table 3. The detailed information for the free energy change of reaction intermediates (*OH, *O, *OOH) on the unsaturated Ru site.

Structure	Model	E/eV	G(T)/eV	G	Process	G-final	G (U=0 V)	ΔG
**RuO_2**	Slab	-1343.076	0	-1343.076	$2H_2O$	-1371.510	0	
	*OH	-1353.752	0.320	-1353.432	$H_2O + 1/2\ H_2$	-1371.055	0.455	0.455
	*O	-1349.038	0.050	-1348.988	$H_2O + H_2$	-1370.016	1.494	1.039
	*OOH	-1358.144	0.373	-1357.771	$3/2\ H_2$	-1367.988	3.522	2.028
	Slab	-1343.076	0	-1343.076	$2\ H_2 + O_2$	-1366.590	4.920	1.398
**Si-RuO_2**	Slab	-1394.780	0	-1394.780	$2H_2O$	-1423.215	0	
	*OH	-1405.598	0.335	-1405.263	$H_2O + 1/2\ H_2$	-1422.886	0.329	0.329
	*O	-1400.770	0.054	-1400.716	$H_2O + H_2$	-1421.744	1.470	1.141
	*OOH	-1410.033	0.374	-1409.659	$3/2\ H_2$	-1419.876	3.338	1868
	Slab	-1394.780	0	-1394.780	$2\ H_2 + O_2$	-1418.295	4.920	1.582

Supplementary Table 4. Comparison of the OER performance of reported representative Ru-based oxide electrocatalysts in acidic media.

Catalyst	Overpotential (mV@10 mA cm^{-2})	Tafel slope	Stability (h) @10 mA m^{-2}	Degradation rate (μV h^{-1}) *	Reference
**Si-RuO_2-0.1**	**226**	**33**	**800**	**52**	**This work**
$W_{0.2}Er_{0.1}Ru_{0.7}O_{2-\delta}$	168	66.8	500	176	[1]
S-RuCuO NRs/C	231	39.7	250	176	[2]
PRPO-350	174	28.8	150	385	[3]
Ru_3MoCeO_x	164	61.2	100	194	[4]
$Li_{0.52}RuO_2$	156	83.5	70	1685	[5]
a/c RuO_2	205	48.6	60	1450	[6]
$Co_{0.11}Ru_{0.89}O_2$	169	49	50	1580	[7]
C-RuO_2-RuSe-10	242	50.4	50	3840	[8]
Mg-RuO_2	228	48.66	30	4666	[9]
$CaCu_3Ru_4O_{12}$	171	40	24	875	[10]
75-H-RuO_2	200	71	20	3950	[11]
Ufd-RuO_2/CC	179	36.9	20	950	[12]
$Mn_{0.73}Ru_{0.27}O_2$	208	65.3	10	5200	[13]
Mn-RuO_2	158	42.94	10	19200	[14]
$Cr_{0.6}Ru_{0.4}O_2$	178	58	10	5600	[15]
RuO_2-WC NPs	347	88.5	10	2800	[16]
Cu-doped RuO_2	188	43.96	8	10375	[17]
RuO_2 NSs	199	38.2	~6.9	5940	[18]
YBRO-0.15	278	40.8	5	4583	[19]
$Y_{0.85}Mg_{0.15}Ru_2O_7$	258	39.1	5	1301	[20]

*The degradation rate was calculated from the reported chronopotentiometry curves under a constant current density of 10 mA cm^{-2}.

Supplementary Note 1

The effective ionic radii and bond dissociation energies were obtained Lange's Handbook of Chemistry (15th Ed.)

Supplementary References

1. Hao, S., Liu, M., Pan, J., Liu, X., Tan, X., Xu, N., He, Y., Lei, L., Zhang, X. Dopants fixation of Ruthenium for boosting acidic oxygen evolution stability and activity. *Nat. Commun.* **11** 5368 (2020).
2. Yao, Q., Yu, Z., Chu, Y.-H., Lai, Y.-H., Chan, T.-S., Xu, Y., Shao, Q., Huang, X. S incorporated RuO_2-based nanorings for active and stable water oxidation in acid. *Nano Res.* **15** 3964-3970 (2022).
3. Ping, X., Liu, Y., Chen, S., Ran, N., Zheng, L., Wang, M., Guo, L., Wei, Z. Tailoring B-site of lead-ruthenate pyrochlore for boosting acidic water oxidation activity and stability. *Appl. Catal. B Environ.* **318** 121884 (2022).
4. He, J., Li, W., Xu, P., Sun, J. Tuning electron correlations of RuO_2 by co-doping of Mo and Ce for boosting electrocatalytic water oxidation in acidic media. *Appl. Catal. B Environ.* **298** 120528 (2021).
5. Qin, Y., Yu, T., Deng, S., Zhou, X.-Y., Lin, D., Zhang, Q., Jin, Z., Zhang, D., He, Y.-B., Qiu, H.-J., He, L., Kang, F., Li, K., Zhang, T.-Y. RuO_2 electronic structure and lattice strain dual engineering for enhanced acidic oxygen evolution reaction performance. *Nat. Commun.* **13** 3784 (2022).
6. Zhang, L., Jang, H., Liu, H., Kim, M.G., Yang, D., Liu, S., Liu, X., Cho, J. Sodium-Decorated Amorphous/Crystalline RuO_2 with Rich Oxygen Vacancies: A Robust pH-Universal Oxygen Evolution Electrocatalyst. *Angew. Chem. Int. Ed.* **60** 18821-18829 (2021).
7. Tian, Y., Wang, S., Velasco, E., Yang, Y., Cao, L., Zhang, L., Li, X., Lin, Y., Zhang, Q., Chen, L. A Co-Doped Nanorod-like RuO_2 Electrocatalyst with Abundant Oxygen Vacancies for Acidic Water Oxidation. *iScience* **23** 100756 (2020).
8. Wang, J., Cheng, C., Yuan, Q., Yang, H., Meng, F., Zhang, Q., Gu, L., Cao, J., Li, L., Haw, S.-C., Shao, Q., Zhang, L., Cheng, T., Jiao, F., Huang, X. Exceptionally active and stable RuO_2 with interstitial carbon for water oxidation in acid. *Chem* **8** 1673-1687 (2022).
9. Li, Z., Wang, S., Tian, Y., Li, B., Yan, H.j., Zhang, S., Liu, Z., Zhang, Q., Lin, Y., Chen, L. Mg-Doping improves the performance of Ru-based electrocatalysts for the acidic oxygen evolution reaction. *Chem. Commun.* **56** 1749-1752 (2020).
10. Miao, X., Zhang, L., Wu, L., Hu, Z., Shi, L., Zhou, S. Quadruple perovskite ruthenate as a highly efficient catalyst for acidic water oxidation. *Nat. Commun.* **10** 1-7 (2019).
11. He, J., Chen, W., Gao, H., Chen, Y., Zhou, L., Zou, Y., Chen, R., Tao, L., Lu, X., Wang, S. Tuning hydrogen binding modes within RuO_2 lattice by proton and electron co-doping for active and stable acidic oxygen evolution. *Chem Catal.* **2** 578-594 (2022).
12. Ge, R., Li, L., Su, J., Lin, Y., Tian, Z., Chen, L. Ultrafine Defective RuO_2 Electrocatayst Integrated on Carbon Cloth for Robust Water Oxidation in Acidic Media. *Adv. Energy Mater* **9** 1901313 (2019).
13. Wang, K., Wang, Y., Yang, B., Li, Z., Qin, X., Zhang, Q., Lei, L., Qiu, M., Wu, G., Hou, Y. Highly active ruthenium sites stabilized by modulating electron-feeding for sustainable acidic oxygen-evolution electrocatalysis. *Energy Environ. Sci.* **15** 2356-2365 (2022).
14. Chen, S., Huang, H., Jiang, P., Yang, K., Diao, J., Gong, S., Liu, S., Huang, M., Wang, H., Chen, Q. Mn-Doped RuO_2 Nanocrystals as Highly Active Electrocatalysts for Enhanced Oxygen Evolution in Acidic Media. *ACS Catal.* **10** 1152-1160 (2020).
15. Lin, Y., Tian, Z., Zhang, L., Ma, J., Jiang, Z., Deibert, B.J., Ge, R., Chen, L. Chromium-ruthenium oxide solid solution electrocatalyst for highly efficient oxygen evolution reaction in acidic media. *Nat. Commun.* **10** 162 (2019).
16. Sun, S.-C., Jiang, H., Chen, Z.-Y., Chen, Q., Ma, M.-Y., Zhen, L., Song, B., Xu, C.-Y. Bifunctional

WC-Supported RuO_2 Nanoparticles for Robust Water Splitting in Acidic Media. *Angew. Chem. Int. Ed.* **61** e202202519 (2022).

17. Su, J., Ge, R., Jiang, K., Dong, Y., Hao, F., Tian, Z., Chen, G., Chen, L. Assembling Ultrasmall Copper-Doped Ruthenium Oxide Nanocrystals into Hollow Porous Polyhedra: Highly Robust Electrocatalysts for Oxygen Evolution in Acidic Media. *Adv. Mater.* **30** 1801351 (2018).
18. Zhao, Z.L., Wang, Q., Huang, X., Feng, Q., Gu, S., Zhang, Z., Xu, H., Zeng, L., Gu, M., Li, H. Boosting the oxygen evolution reaction using defect-rich ultra-thin ruthenium oxide nanosheets in acidic media. *Energy Environ. Sci.* **13** 5143-5151 (2020).
19. Feng, Q., Zou, J., Wang, Y., Zhao, Z., Williams, M.C., Li, H., Wang, H. Influence of Surface Oxygen Vacancies and Ruthenium Valence State on the Catalysis of Pyrochlore Oxides. *ACS Appl. Mater. Interfaces* **12** 4520-4530 (2020).
20. Feng, Q., Zhang, Z., Huang, H., Yao, K., Fan, J., Zeng, L., Williams, M.C., Li, H., Wang, H. An effective strategy to tune the oxygen vacancy of pyrochlore oxides for electrochemical energy storage and conversion systems. *Chem. Eng. J.* **395** 124428 (2020).
